# Diastereomeric resolution directed towards chirality determination focussing on gas-phase energetics of coordinated sodium dissociation

**DOI:** 10.1038/srep24005

**Published:** 2016-04-04

**Authors:** Osamu Kanie, Yuki Shioiri, Koji Ogata, Waka Uchida, Shusaku Daikoku, Katsuhiko Suzuki, Shinichiro Nakamura, Yukishige Ito

**Affiliations:** 1 Tokai University, Institute of Glycoscience, 4-1-1 Kitakaname, Hiratsuka, Kanagawa 259-1292 Japan; 2 Tokyo Institute of Technology, Graduate School of Bioscience and Biotechnology, 4259 Nagatsuta, Midori-ku, Yokohama 226-8503 Japan; 3 RIKEN, Synthetic Cellular Chemistry Laboratory, 2-1 Hirosawa, Wako, Saitama 351-0198 Japan; 4 RIKEN, Research Cluster for Innovation, 2-1 Hirosawa, Wako, Saitama 351-0198 Japan; 5 Japan Science and Technology Agency (JST), ERATO Glycotrilogy Project, 2-1 Hirosawa, Wako, Saitama 351-0198 Japan; 6 Advanced Center for Computing and Communication, Computational Chemistry Unit, 2-1 Hirosawa, Wako, Saitama 351-0198 Japan

## Abstract

Defining chiral centres is addressed by introducing a pair of chiral auxiliary groups. Ions of diastereomeric pairs of molecules could be distinguished utilising energy-resolved mass spectrometry, and the applicability of the method to a series of compounds carrying amine, carboxylic acid, alcohol, and all the amino acids was verified. The method was further strengthened by distinguishing diastereomeric ions that did not undergo fragmentation. Mass spectrometric evaluation of the dissociation process of adducted sodium cations from the diastereomeric precursors agreed with the theoretical calculations, indicating the potential usefulness of the method for the determination of absolute configurations.

Chiral recognition events occur ubiquitously among living things. Vast numbers of such recognition events can be observed in enzyme–substrate and receptor–counter-receptor interactions, and they form the foundations of life. For example, the natural product (*R*)-(−)-1-octen-3-ol imparts a characteristic mushroom flavour to food^1^,^2^, whose sensing by the human olfactory receptor protein is initiated by the intermolecular chiral–chiral recognition event. The discrimination and determination of the absolute configurations of chiral centres are also of particular importance in organic chemistry, biochemistry, and analytical chemistry. Although mass spectrometry (MS) is often used for the structural analysis of a wide range of compounds, it is known to be ‘chiral-blind’, like many other analytical methods. MS is used to determine mass-to-charge ratios (*m/z* values), which relate to the molecular structure of an analyte, and it can further provide partial structure information through tandem MS experiments^3^,^4^. If the MS technique could be extended to resolve the chirality of an ion generated from a molecule, its importance would be immeasurably increased.

In analytical chemistry, chirality can only be directly defined by methods based on optical phenomena, such as the observation of optical rotation, the Bijvoet method in X-ray crystallography^5^, and the recently reported Coulomb explosion imaging approach^6^. Other commonly used methods to determine chirality rely on intermolecular chiral–chiral interactions, or involve the analysis of diastereomeric pairs after derivatisation. Unlike enantiomers, diastereomers have different chemical properties, allowing the use of a wider range of analytical techniques and affording greater convenience^7^. A drawback in the analysis of diastereomers is the requirement of an extra derivatisation step, after which the isolated derivatives can be analysed. Mosher’s method, which uses nuclear magnetic resonance (NMR) spectroscopy to evaluate the magnetic anisotropy introduced by a chiral auxiliary group, is used to determine the absolute configuration of chiral compounds^8^.

Several MS-based methods capable of resolving isomeric ions are known. Techniques based on the analysis of complexes of chiral guests and chiral hosts have been reported, which provide information regarding the kinetics of association or dissociation of non-covalent complexes^9^,^10^,^11^,^12^,^13^,^14^,^15^,^16^,^17^,^18^. Ion mobility MS has been used as a platform, with the aid of a chiral neutral gas, to differentiate the drift times of ionised chiral molecules^19^. These methods often require a specific partner, limiting their generality. At the same time, such methods are advantageous for distinguishing diastereomers because common ion species such as proton and sodium adducts can be conventionally handled. The discrimination of diastereomeric pairs of small peptides based on collision-induced dissociation (CID) has been reported, in which the product ions generated from the corresponding precursor ions and their signal intensities were investigated^20^,^21^,^22^,^23^. Furthermore, the usefulness of energy-resolved mass spectrometry (ERMS) has been shown for distinguishing isomeric ions of a wide range of molecules^17^,^22^,^24^,^25^,^26^,^27^,^28^. Despite its potential for the analysis of diastereomeric ions, the generality of this method has not been assessed. Another problem with CID is that no information can be obtained about the precursor ion when the metal cation adduct dissociates before the breakdown of other constituent chemical bonds.

In the course of our investigations to develop a new method for the analysis of glycan structures, we reported that the anomeric configurations of carbohydrates, which may be considered as examples of diastereomers, could be determined by ERMS^29^,^30^,^31^. Encouraged by the fact that these closely related diastereomers could be easily resolved by ERMS, we endeavoured to further resolve chiral compounds after derivatisation by introducing a chiral auxiliary group by focusing on the activation energy under low-energy CID conditions to show applicability of ERMS method for structural determination.

Herein, we describe a method for determining the absolute configuration of chiral compounds based on MS, focusing on the activation energy differences between the sodium adducts of diastereomeric pairs. The following were the important objectives of this study: (1) to confirm applicability of ERMS method to a wide range of diastereomers derived from a pair of chiral compounds; (2) to distinguish a pair of isomeric ions derived from small molecules that do not produce fragment ions; and (3) to understand the principles underlying the discrimination. The ERMS technique was able to discriminate between a series of diastereomeric molecular ion pairs containing chiral auxiliaries, which suggested that the method could be applied to a wide range of compounds. Furthermore, even small ions that preferentially lost sodium cations under low-energy CID conditions leading to a total loss of molecule-oriented ions in the spectra could be distinguished from one another. Finally, a theoretical basis was suggested to explain the phenomena.

## Results

In the analysis of chiral molecules, target compounds are often derivatised into diastereomers by introducing a chiral auxiliary group. An enantiomeric *R*- and *S*- pair of target compounds provides a pair of diastereomers, RR*- and SR*-isomers, after the introduction of an auxiliary group (R*) with defined chirality. According to previous reports^20^,^28^, the ions generated from these diastereomers might be generally distinguishable using MS. When only one of the compounds is available, e.g., the *R*-isomer of a pair of enantiomers, a pair of chiral auxiliary groups can be introduced, resulting in RR*- and RS*-isomers, which are also distinguishable. Subtle energy differences in CID have to be considered in the analysis of these diastereomeric isomers. For this reason, low-energy CID conditions are suitable for the analysis of such isomeric ions.

### Decay analysis of all combinations of *R*/*S*-valine and *R*/*S*-proline

**Figure 1:**
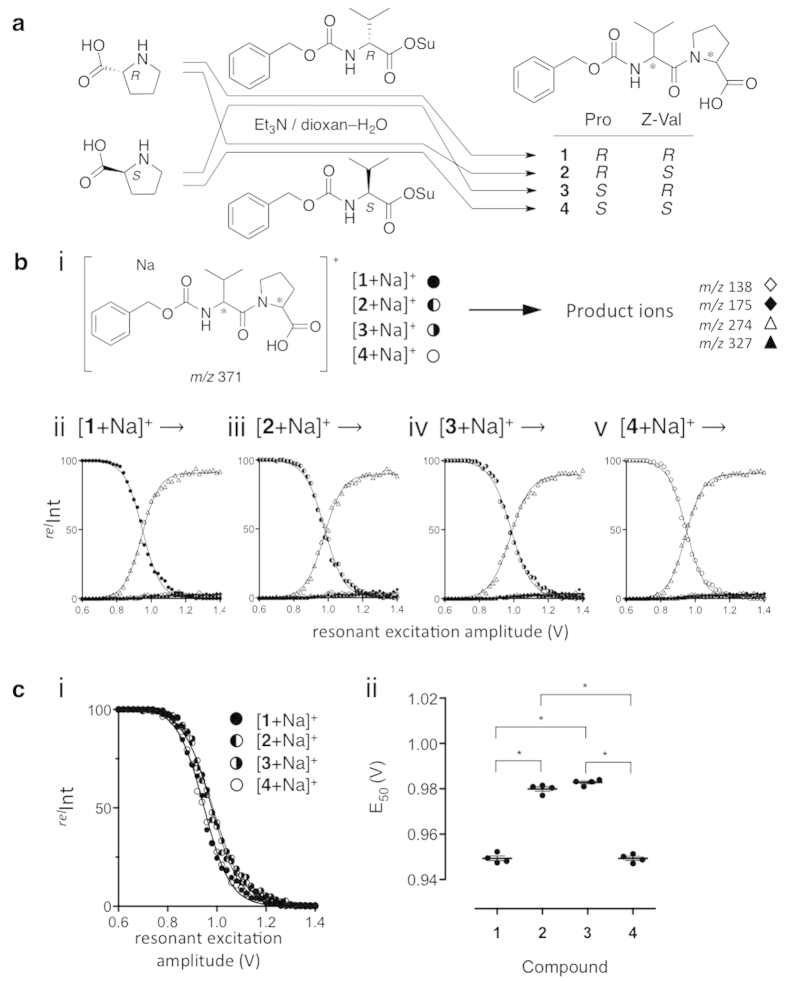
Derivatisation and ERMS analysis of dipeptide diastereomers. (**a**) *R*- and *S*-proline are derivatised into compounds **1**–**4** consisting of enantiomers and diastereomers by introducing a pair of chiral auxiliary groups. (**b**) (i) Gas-phase dissociation reaction of the set of dipeptides based on ERMS. See Supplementary Fig. S2 for structures of individual product ions. (ii–v) ERMS diagrams of sodiated compounds **1**–**4** (**c**) (i) Overlay of break down curves of sodiated compounds **1**–**4**. (ii) Scattered plot of E_50_ values of the series of compounds. E_50_ values were obtained by global non-linear regression analysis using the Boltzmann equation (*n* = 4) where mean and the standard error of the obtained E_50_ were shown. The asterisk indicates significance with P < 0.05.

To examine the above hypothesis, all four possible d- and l-proline derivatives ((*R*)- and (*S*)-Pro, respectively) containing chiral auxiliary groups were synthesised and analysed by ERMS. All combinations of the dipeptides with SS*-, SR*-, RS*-, and RR*-configurations were prepared by the coupling of *R*- and *S*-Pro with the succinimidyl esters of benzyloxycarbonyl (Z)-protected R*- and S*-valine (Val) (Fig. 1a). Tandem mass spectrometric analysis provided a series of product ions that showed only slight differences in the signal heights between the diastereomeric pairs, as anticipated (Fig. 1b). All of the Na^+^ adducted precursor ions (*m/z* 371) dissociated similarly to produce product ions with *m*/*z* 138, 175, 274, and 327. The decay curves in Fig. 1b(ii–iv), however, could be classified into two groups (Fig. 1c (i)). The half-values of the activation energies (E_50_) required for the dissociation of a precursor ion were obtained from the ERMS analyses of these compounds. Finally, it is clear that, although the E_50_ values of the enantiomers are statistically indistinguishable, those of the diastereomers are distinguishable (Fig. 1c(ii)).

### Resolution of a variety of compounds containing different functional groups using E_50_ values

**Figure 2:**
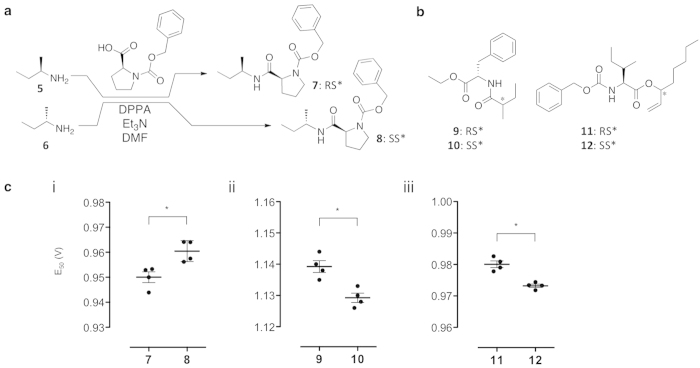
Derivatisation and ERMS analysis of a chiral amine, acid, and alcohol. (**a**) Derivatisation of the enantiomers of 1-methylpropylamine. (**b**) Structures of diastereomeric pairs of 2-metylbutyric acid and 1-octen-3-ol derivatives. (**c**) Scattered plot of E_50_ values obtained for sodiated compounds (i) **7** and **8**, (ii) **9** and **10**, and (iii) **11** and **12**. Individual E_50_ values were obtained by global non-linear regression analysis using the Boltzmann equation (*n* = 4) where mean and the standard error of the obtained E_50_ were shown. The asterisk indicates significance with P < 0.05.

Having shown that the dipeptide diastereomers can be distinguished, the applicability of the discrimination method was further investigated. Thus, the diastereomeric pair (**7** and **8**) of the simple compounds (*R*/*S*)-1-methylpropylamine (**5** and **6**), a synthetic chiral intermediate, containing Z-(*S*)-Pro as a chiral auxiliary group was prepared (Fig. 2a). The diastereomeric derivative sets of natural products, namely (*R*/*S*)-2-methylbutyric acid (**9** and **10**) and (*R*/*S*)-1-octen-3-ol (**11** and **12**) were also prepared (Fig. 2b). The sodium adducts of these compounds were analysed by ERMS, from which the E_50_ values were obtained by measuring the decay processes under CID conditions, which clearly distinguished the pairs of diastereomers (Fig. 2c). The only difference in each set of compounds was the single instance of chirality at the carbon atoms bonded to the functional groups.

**Figure 3:**
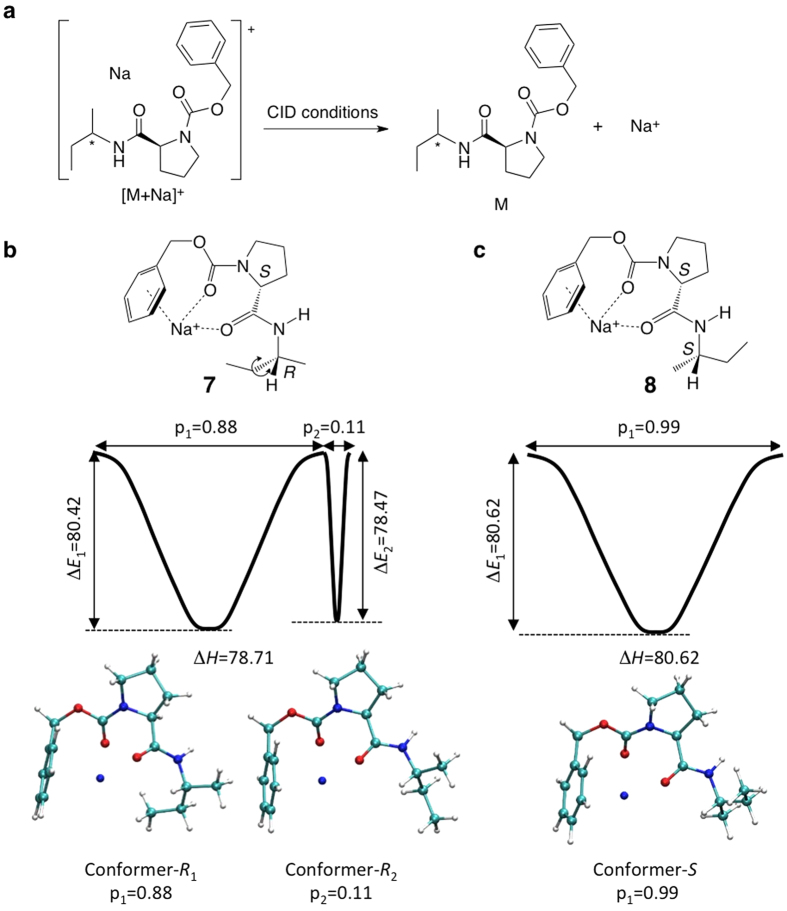
Density functional theory calculations and theoretical basis for the observed discrimination. (**a**) Gas-phase dissociation of Na^+^ from adducts **7** and **8**. Δ*E*_*a*_ analysis for conformers based on theoretical calculations of compounds (**b**) [**7** + Na]^**+**^ and (**c**) [**8** + Na]^+^.

It should be noted that the dissociation of the sodium cation from the precursor ions was the only detectable event under the low-energy dissociation conditions in the cases of compounds **7**, **8**, **9**, and **10** (Fig. 2b). (See Discussion for possible precursor loss) The current ERMS method, which analyses the decay of a precursor ion, can distinguish a diastereomeric pair even when no fragmentation is observed. Here, the dissociation of the metal cation adduct itself is considered as part of the fragmentation reaction under the given low-energy CID conditions (Fig. 3a). The decay of a metal-adducted precursor ion over a range of CID energies represents not only the result of fragmentation, but also dissociation of the metal cation in general. As shown in Fig. 2c(i), the sodium adduct of compound **7** (RS*), [M + Na]^+^, requires lower CID energy than compound **8** (SS*). Since no fragmentation is observed, the difference in E_50_ values was attributed to the lower affinity of compound **7** towards the sodium cation (Fig. 3a).

### Theoretical explanation of the discrimination data obtained by MS

We attempted to develop a successful methodology for determining the stereochemistry of diastereomers by assessing its applicability and laying theoretical groundwork. We sought an explanation for the observed differences in energy required for sodium dissociation by performing theoretical calculations for compounds **7** and **8**, which did not produce any product ions, to show the method’s potential for the determination of absolute configuration. Decay curves obtained in the MS experiments are associated with the dissociation of sodium cations from sodiated diastereomeric molecules; thus, the energy required for the release of the sodium cation was investigated by density functional theory. Unlike solution chemistry, the activation energy leading to the transition state determines the non-equilibrium gas-phase dissociation reaction. To compare the difference in energies of the diastereomeric ions, the possible conformers involved in each step must be considered. In the current study, we considered the energy required for sodium dissociation, namely 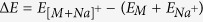, as a substitute for the activation energy, where *E* is the energy of the individual entities. Structural features obtained from the calculations show that: 1) the sodium atom is coordinated to two carbonyl groups and the π electrons of the benzene ring; 2) the overall conformations of both diastereomers are very similar; 3) the two major conformers share the aforementioned scaffold for both compounds, which are the rotational isomers around the bond between the amine function and the α-carbon (conformers-*R*_1_, -*R*_2_, and -*S* in Fig. 3b,c); and 4) minor conformational variations are associated with the rotation of the bond between the α- and β-carbons (indicated by the arrows in Fig. 3b). Although one of the conformers (*R*_1_) of sodiated compound **7** was the most stable, Δ*H* for compound **7** was almost equal to that of sodiated compound **8** when the frequency of the appearance of the conformers was considered. The calculated average Δ*H* for sodiated **7** and **8** were 78.71 and 80.62 kcal/mol, respectively, indicating that compound **7** had a slightly lower affinity towards Na^+^. Thus, the obtained simplified energies required for sodium dissociation were in agreement with the experimental behaviour of the sodiated molecules under CID conditions.

## Discussion

Although the diastereoresolution methods based on chiral HPLC and MS are conceptually similar, the latter method, which handles gas-phase reactions, is considered to fit better with theoretical approaches because of the exclusion of solvent interactions. The requirement of only a small amount of analyte is also advantageous.

To distinguish structurally closely related compounds under CID conditions, subtle energy differences have to be analysed. Thus, low-energy CID conditions are suitable for the analysis of such isomeric ions. During the course of our investigation to analyse anomeric configurations of glycosidic bonds, we examined ERMS. Since the energy input, provided by the repeated collision of an ion and an inert gas under CID conditions, is the governing factor in the dissociation reactions, the precise control of collision energy should resolve the structurally closely related molecular ion species and fragment ions produced during the low energy CID process.

The ERMS analyses of all combinations of *R/S*-Pro connected to Z-protected *R/S*-Val as the chiral auxiliary were carried out, and the results are shown in Fig. 1. It can be clearly seen from the E_50_ values of the ERMS decay curves that successful discrimination of a pair of diastereomers was achieved, despite the fact that the commonly used MS/MS did not provide sufficient data to distinguish these isomers (Supplementary Fig. S2). Such MS/MS experiments provided information supporting the structures of the precursor ions; however, the observed product ions were almost identical, with very small intensity differences. The ERMS experiments also clearly indicated that the enantiomers could not be discriminated. This is of particular importance, because the hypothetical mass spectrometric data for a compound, SR*, even when it is not available, can be obtained from the mirror-image RS* compound. This might be useful in the real world identification of chiral compounds; furthermore, the absolute structure might be obtained, since the data set for the diastereomeric pair is obtained even when one does not have one of the enantiomers at hand. The results suggest that the data for the individual enantiomers of proline can be obtained, even when only one enantiomer is available, as we initially hypothesised.

The applicability of the discrimination method was then investigated for a series of compounds containing different functional groups. Thus, the behaviour of the diastereomeric pairs of the simple compounds (*R*/*S*)-1-methylpropylamine (**7** and **8**), (*R*/*S*)-2-methylbutyric acid (**9** and **10**), and (*R*/*S*)-1-octen-3-ol (**11** and **12**) containing individual chiral auxiliary groups was examined (Fig. 2). All pairs of compounds were distinguishable regardless of the functional group, which indicated that a wide range of chiral compounds containing a variety of functional groups could be distinguished by this method. In addition, all of the d- and l-amino acids were investigated and successfully resolved (see the Supplementary Fig. S1). These results indicated the versatility of the diastereoresolution technique, based on ERMS. It should be stated here about a question regarding day-to-day variation of the ERMS data because the exact CID conditions are not reproducible. Although it is true that exact E_50_ values are not obtainable, our data indicates the relative relationship that determines the stability difference of a pair of diastereomeric ions remains (see the Supplementary Fig. S3).

The loss of adducted metal ions is one of the fundamental problems in the analysis of the dissociation reactions of ions generated from relatively small molecules (i.e., Mw ≤ 300) comprising relatively stable chemical bonds. We have to consider a possible precursor loss due to the high-energy input resulting in resonant ejection, because we cannot observe any product ions but decreasing precursor ion. However, this is unlikely occurring in the current experimental setup considering that the relationship of applied end-cap amplitude and *m/z* value of all the analysed 46 ions throughout the experiments (*m/z* range: 300.2–460.2 and E_50_ range: 0.95–1.25 V) regardless of the presence or absence of fragmentation pathway. We therefore tentatively consider the observed decay of the precursor ion during ERMS study in these cases reflects the dissociation of adducted sodium cation from precursor, which is also supported by the observation of sodium cation in the MS/MS of sodiated galactose^32^. Since the MS technique typically analyses ions, information regarding partial structure cannot be obtained. Gradually increasing the CID energy in an effort to obtain product ions often results in the loss of metal adducts from the precursor ions. Considering that many biomolecules are small and some are stable, it would be highly desirable to develop an analysis method that can assess their stereochemical configurations. The dissociation of the metal cation adduct itself is considered a part of the fragmentation reaction under the given low-energy CID conditions. Generally, the decay of a metal-adducted precursor ion over a range of CID energies represents not only the result of fragmentation, but also the dissociation of the metal cation. This suggests that ERMS analysis that focuses on the decay of a precursor ion can be useful, especially for cases in which no obvious fragmentation reactions occur. In fact, the dissociation of the sodium cation from the precursor ions was the only detectable event for compounds **7**, **8**, **9**, and **10** (Fig. 2c). In the current study, Na^+^ could not be observed due to the low mass cut-off (LMC) in the quadrupole ion trap (QIT) MS that may be seen as a disadvantage. This might be overcome, in the future, by analysing cesium adducted ions, because 1) LMC can be avoided up to molecular weight with ca 400 and 2) cesium tends to dissociate faster than fragmentation reactions under CID conditions as it was discovered in the analysis of metal adducts of glycans^32^,^33^,^34^. Non-ion trap Q type MS can also be a good candidate because it covers wider *m/z* range when one focuses on metal dissociation^34^.

Theoretical analysis may offer deeper understanding in the development of a methodology for determining the stereochemistry of diastereomeric compounds. Theoretical calculation is easier for gas-phase chemistry; therefore, the current format is suitable. As we discussed, the loss of sodium from an ion is one of the dissociation reactions, and we therefore rationalised the evaluation of sodium dissociation. As shown in Fig. 2c(i), sodiated RS* compound **7** requires lower CID energy than compound **8** (SS*). The observed difference in E_50_ values reflected the affinity of compounds toward the sodium cation (Fig. 3a). It is considered that the sodiated molecule reaches a transition state after excitation through repeated collisions with the inert helium gas, and the coordinated sodium cation dissociates to generate the corresponding neutral molecule and sodium cation, although adducted sodium could not be observed due to the spectrometer’s limitation. The energy for the release of the sodium cation was investigated by density functional theory. In the gas-phase dissociation reaction, the activation energy determines the reaction pathway because of the non-equilibrium nature. After iterative calculations, it was found that the average Δ*H* for sodiated **7** (78.71 kcal/mol) and **8** (80.62 kcal/mol) were different, indicating that sodiated **7** would dissociate at lower energy. This result is consistent with the ERMS experimental observations. Considering the CID process involves approximately 10^6^ ions, the ERMS results reflect various conformers. The results suggested that the theoretical calculations estimated the ionic stability differences and that MS provided experimental confirmation; therefore, the absolute configuration of a chiral centre can be determined by focusing on the energy required for sodium dissociation in the case of small and stable ions with the aid of a chiral auxiliary.

Furthermore, we described above that data for a compound that does not exist could be obtained from its mirror-image compound, based on the introduction of a pair of chiral auxiliary groups. This has practical importance when only one of the enantiomers is available. Since it was shown for derivatives of (*R*/*S*)-2-methylbutyric acid (**9** and **10**) carrying Z-protected l(*S*)-proline coud be discriminated by MS and was supported by calculations, discrimination of *R*/*R* vs. *R*/*S* is also possible. Therefore, the chirality of the **9** if it is not identified is possible. The advantage of using MS in analysis is that a target ion can be ‘isolated’ according to its *m/z* value prior to MS/MS analysis. As a result, a post-derivatisation purification process is not necessary (Supplementary Table S1). Moreover, sodium adducts, which are frequently observed and available to many researchers, were used to differentiate the diastereomeric ions.

## Conclusion

The elucidation of chirality is of fundamental importance in chemistry. Despite the fact that mass spectrometry is advantageous when the amount of sample available for analysis is limited, the technique is known to be ‘chiral-blind’. To address this issue, we 1) discriminated the ionised small molecules containing a single chiral centre after derivatisation into a pair of diastereomers; 2) carried out the analysis of compounds that do not undergo any fragmentation reactions by analysing the dissociation of the sodium cation from a precursor ion; and 3) provided a theoretical explanation of the differences observed for sodium dissociation in the mass spectrometric analysis. In this manner, it was indicated that diastereomers derived from a chiral molecule by introducing a pair of chiral auxiliary group could be discriminated by using mass spectrometry, which was supported by theoretical calculations. The method of analysing the decay process of a pair of diastereomeric precursor ions was performed on a quadrupole ion-trap mass spectrometer where a potential problem of low mass cut could be avoided if one uses non-ion trap mass spectrometer. In the current experimental setup, relative stability difference between diastereo-pairs is obtained, however the introduction of normalisation method may further enhance the utility of the method. The use of external energy reference would be a practical candidate.

## Methods

### General methods

Amino acids and protected amino acids were purchased from PEPTIDE INSTITUTE, Inc. (Osaka, Japan). Acetonitrile (HPLC grade) for LC analysis was purchased from Wako Pure Chemical Industries, Ltd. (Osaka, Japan). Trifluoroacetic acid (TFA) was purchased from Thermo Fisher Scientific (Waltham, MA). Water was purified by a Milli-Q apparatus (Millipore Co., Milford, MA). HPLC grade solvents were used for mass spectrometric analysis; MeOH and CHCl_3_ (Wako Pure Chemical Industries, Ltd, Osaka, Japan).

The compounds were separated using a Unison-UK C-18 column (250 mm × 4.6 mm i.d.) (Imtakt, Co., Kyoto, Japan). Diastereomers derived from amines were separated using CHIRALPAK IA (250 mm × 4.6 mm i.d.) (Daicel Co., Osaka, Japan), when needed. An isocratic or a linear gradient elution was applied at a flow rate of 1.0 mL/min at 40 °C using water containing 0.1% TFA (solvent A) and acetonitrile containing 0.1% TFA (solvent B).

HPLC apparatus used in this study consisted of Waters 2767 Sample Manager as the injector, Waters 515 HPLC Pump as the makeup pump, Waters 2525 Binary Gradient Pump, Waters 490E Programmable Multiwavelength Detector (λ = 254 nm) (Waters Co., Milford, MA), and SSC-2120 Column Oven (Senshu Scientific Co., Ltd., Tokyo, Japan). QIT-MS was also used as a detector (see below for details).

### Mass spectrometry instrumentation and data collection

Samples were analysed using a quadrupole ion-trap mass spectrometer (QIT-MS) coupled with an electrospray interface (Esquire 3000^+^, Bruker Daltonics GmbH, Bremen, Germany). Samples dissolved in MeOH (0.002–0.02 μmol/mL) were introduced into the ion source *via* infusion (flow rate, 120 μL/h). The parameters for analysis were: (1) ‘drying temperature’: 250 °C, (2) nebulizer gas (N_2_): 10 psi, (3) drying gas (N_2_): 4.0 L/min, (4) ‘Smart frag’: off, (5) scan range: *m*/*z* 50–600 or 700, (6) compound stability: 300%, (7) ICC target: 5,000, (8) maximum acquisition time: 200 ms, (9) average: 10 spectra, (10) ‘cut-off’: 27.6% and (11) collision gas: He (6.0 × 10^−6^ mbar).

In our MS^*n*^ experiments, the end-cap radio frequency (r.f.) amplitude was raised by 0.02 V increments until the precursor ion could no longer be detected (plateau at less than 0.9% of total ion current). Averages of *m* − 4 spectra were used for CID experiments (*m* = 9–15, where *m* is the number of spectra obtained during the experiments); the first and last two datasets, which are associated with a transient period to steady state in an r.f. amplitude step, were not used to avoid inaccuracies.

Isotopic peaks with [I^*i*^ + 1] and [I^*i*^ + 2], where I^*n*^ indicated a fragment ion, were included in the calculations (refer to Data Handling). For the isolation of a product ion, *m*/*z* ± 2 (range of isotopes, *w* = 2) were isolated and subjected to the CID experiments to include isotopes. In such a case, we set *w* = 2; thus, *m*/*z* ± 0.8 were isolated to exclude unintended isotopes. Standard MS/MS spectra are the extracts of these ERMS at designated amplitudes.

### Mass spectrometrical data handling

In order to obtain graphs of ERMS, the following equations were used. When an ion ‘I^*P*^’ produces a series of product ions I^1^, I^2^, I^3^, …I^*i*^, the relative ion currents for individual ions were defined by equation 1,


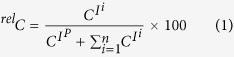


where ^*rel*^*C* indicates the ion current of a given ion among the observed ions in per cent, 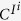 is the observed ion current in focus, and 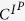 is the ion current of a precursor ion. The calculations were performed using a program developed by us with Excel (Excel 2000 (Microsoft Corp.)), which was based on a DSUM function and programmed to choose a range of isotopes (*w*) to be taken into consideration (*w* = 2 in the experiments).

### Sigmoidal curve fitting of ERMS data

A set of MS^*n*^ data obtained at various r.f. end-cap amplitudes on a mass spectrometer was analysed using Excel, where peak sets with certain *m*/*z* values were treated as a series of data. The relative intensities over total ion current for individual signals were obtained at each amplitude (equation 1). The data were analysed using Prism 4 software (GraphPad Software, Inc.). Individual data for the decay of precursor ions were fitted using the Boltzmann sigmoidal function with non-linear regression analysis (equation 2),


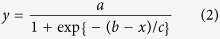


where parameters ‘*a*’, ‘*b*’, and ‘*c*’, which indicate a maximum response, half value of the maximum response (E_50_ in the current study), and a slope factor, respectively, were obtained for each curve. In the series of data used in this report, the sigmoidal curves and parameters were obtained by plotting the regression curves by processing all of the data obtained from the above Excel program.

For analysing the dissociation of compounds that did not produce product ions, the data were not normalised because the only visible ion was the precursor. Thus, the non-regression curve fitting to share *b* and *c* values was carried out to judge the five experimental data sets. After deciding the data sets according to R^2^, global analysis of four individual ERMS experimental data was again carried out to share *c* value to obtain E_50_ (*b*) value. These were carried out using Prism 4 (see the Supplementary Information online for more detail).

### Note for introducing the chiral auxiliary group

The chiral auxiliary was introduced to the target compounds using a succinimidyl activated auxiliary group. It is possible that the ions with different *m/z* values can be simply differentiated by mass spectrometric analysis. Thus, a mixture of compounds may be used without carrying out purification procedures. Based on this consideration, several sets of amino acid mixtures were prepared and subjected to coupling reactions (Supplementary Table S1). The protecting groups were removed by standard conditions when needed, and the formed compounds were analysed by MS directly after workup. The purity of the synthesised diastereomers was confirmed by HPLC analysis. The retention times and analytical conditions are shown in Supplementary Table S2.

### Synthesis of Z-l-Val-Pro (3); (typical procedure)

Z-protected l-valine (Z-l-Val, 14.1 mg, 0.056 mmol), 1-(3-dimethylaminopropyl)-3-ethylcarbodiimide hydrochloride (WSCI∙HCl; 11.8 mg, 0.062 mmol), and *N*-hydroxysuccinimide (HO-Suc, 7.1 mg, 0.062 mmol) were mixed in anhydrous dichloromethane (1 mL), and the solution was stirred under a nitrogen atmosphere at room temperature for 2 h. After the reaction completion was confirmed by TLC (1:1 hexane/ethyl acetate), the reaction mixture was diluted with dichloromethane and extracted with 1 *N* HCl. The organic layer was dried with MgSO_4_ and evaporated to dryness, leaving syrup, which was purified by column chromatography (2:1 hexane/ethyl acetate) to obtain Z-l-Val-O-Suc (16.9 mg, 86.7%). A solution of Z-l-Val-O-Suc (9.9 mg, 0.028 mmol) and d-proline (3.3 mg, 0.028 mmol) in 1,4-dioxane (0.5 mL) and water (0.2 mL) containing triethylamine (0.5 mL) was stirred at room temperature for 1 h. The reaction mixture was poured into 1 *N* HCl, which was extracted with ethyl acetate. The organic layer was dried over Na_2_SO_4_ and the organic solution was concentrated under vacuum to form a syrup, which was purified by column chromatography (4:2:1 ethyl acetate/MeOH/water) to obtain Z-l-Val-Pro (8.7 mg, 87.9%). ESI-MS; [M + H]^+^ 349.2, [M + Na]^+^ 371.2, [M + K]^+^ 387.1, [M-H]^-^ 347.2.

### Theoretical analysis

Theoretical analyses based on molecular mechanics (MM), molecular dynamics (MD), and quantum mechanics (QM) at the B3LYP/cc-pVDZ level for neutral molecules and sodiated compounds, respectively, were carried out. The simplified energy Δ*H* required for sodium dissociation was calculated from the differences in *E* for the ion [M + Na]^+^, molecule M, and Na^+^ as follows:


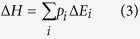


where *p*_*i*_ is the probability of the *i*-th [M + Na]^+^ structure, as given by


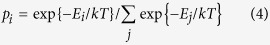


where *E*_*i*_ is the energy of [M + Na]^+^ and *k* and *T* are the Boltzmann constant and temperature, respectively. In equation 3, Δ*E* is the energy difference between [M + Na]^+^ and individual components as follows:


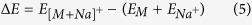


For each conformation, 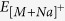, *E*_*M*_, and 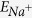 were calculated using QM calculations. To compute Δ*H* for the different entities, the various conformers of each entity forming a complex with Na^+^ were generated by performing MD simulations in a vacuum state using AMBER software package. Before the sampling, the partial charges in the molecules with different conformations were obtained from the optimised structure from B3LYP/6-31G(d) calculations using Gaussian 09. Then, a 2 ns MD simulation for each entity was performed, from which the snapshots were gathered at 10 ps intervals. Finally, 2,000 conformations were extracted from the trajectory of each simulation, and all of the conformations were optimised in the vacuum state using MM calculations. To remove similar conformations, a cluster analysis classified 2,000 optimised structures, with a 1.0 Å rmsd threshold value. In each cluster, the conformation with the lowest energy in the MM calculations was regarded as the representative for the QM calculations. The representative conformations were optimised at the B3LYP/cc-pVDZ level using Gaussian 09. The structures optimised with QM were classified again using cluster analysis, for which the rmsd threshold value was set at 0.5 Å. Finally, 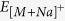 in equation 5 was obtained from the structures with energetically stable conformations in the QM calculations. Analogously, *E*_*M*_ and 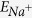 were obtained by single point calculation from the isolated structure generated by removing Na^+^ from the structure of the complex.

## Additional Information

**How to cite this article**: Kanie, O. *et al.* Diastereomeric resolution directed towards chirality determination focussing on gas-phase energetics of coordinated sodium dissociation. *Sci. Rep.* **6**, 24005; doi: 10.1038/srep24005 (2016).

## Supplementary Material

Supplementary Information
