# Supplementary material for: Diastereomeric resolution directed towards chirality determination focussing on gas-phase energetics of coordinated sodium dissociation
Source: Sci Rep. 2016 Apr 4;6:24005. doi: 10.1038/srep24005 (PMC4819175; doi:10.1038/srep24005)
Supplement: Supplementary Information [file srep24005-s1.pdf]

Supplementary Information

Diastereomeric resolution directed towards chirality determination focussing on gas-phase energetics of coordinated sodium dissociation

Authors: Osamu Kanie*, Yuki Shioiri, Koji Ogata, Waka Uchida, Shusaku Daikoku, Katsuhiko Suzuki, Shinichiro Nakamura, and Yukishige Ito

Supplementary materials and methods.

Resolution of D- and L-amino acids. Analysis of the diastereomers of 19 amino acids was carried out to further show the generality of the ERMS-based method. Here, we set up a series of reactions for the introduction of a chiral auxiliary. Since MS is a technique that differentiates ions with different *m/z* values, molecules with different molecular weights can be handled in one-pot (Supplementary Table S1) and were isolated by using HPLC (Supplementary Table S2). A mixture of diastereomers of a series of amino acids containing an auxiliary group is then ionised for MS-analysis.

Supplementary Table S1. Introduction of an auxiliary group to amino acids.

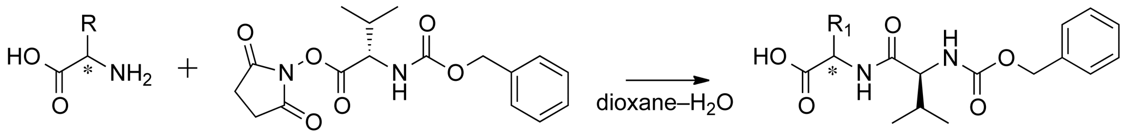

a.a.	a.a. set 1	a.a. set 2	a.a set 3	a.a. set 4	a.a. set 5	a.a set 6	protecting group (deprotection conditions)
Ala	Ala	–	–	–	–	–	–
Arg	–	Arg	–	–	–	–	Pbf (TFA)
Asn	–	–	Asn	–	–	–	–
Asp	–	–	–	Asp	–	–	–
Cys	–	–	–	–	Cys	–	Trt (TFA)
Gln	–	–	Gln	–	–	–	–
Glu	–	–	–	Glu	–	–	–
His	–	–	–	–	His	–	Trt (TFA)
Ile	–	–	–	–	–	Ile	–
Leu	Leu	–	–	–	–	–	–
Lys	–	–	–	–	Lys	–	Boc (TFA)
Met	Met	–	–	–	–	–	–
Phe	–	–	–	–	–	Phe	–
Pro	–	–	–	–	–	Pro	–
Ser	–	–	–	Ser	–	–	–
Thr	–	–	–	Thr	–	–	–
Trp	–	–	–	–	Trp	–	Boc (TFA)
Tyr	–	–	–	Tyr	–	–	–
Val	Val	–	–	–	–	–	–

Supplementary Table S2. Retention time and analytical conditions of synthesised compounds.

Sample name	Retention time (min)		Chiral auxirary	Elution conditions
	R (D)	*S* (L)		
1-Octein-3-ol	53.24	54.34	Z-L-Ile	a
2-Methylbutyric acid	29.91	30.43	L-Phe ethyl ester	b
1-Methylpropylamine	5.17	4.00	Z-L-Val	c
Ala	48.34	47.56	Z-L-Val	d
Arg	40.20	40.36	Z-L-Val	d
Asn	42.84	43.32	Z-L-Val	d
Asp	46.92	47.14	Z-L-Val	d
Cys	54.99	54.38	Z-L-Val	d
Gln	43.46	43.92	Z-L-Val	d
Glu	44.49	44.31	Z-L-Val	d
His	39.51	40.66	Z-L-Val	d
Ile	62.63	60.96	Z-L-Val	d
Leu	60.58	59.87	Z-L-Val	d
Lys	39.52	39.65	Z-L-Val	d
Met	56.03	55.36	Z-L-Val	d
Phe	64.19	62.99	Z-L-Val	d
Pro	54.23	53.87	Z-L-Val	d
Ser	42.51	42.42	Z-L-Val	d
Thr	45.66	44.81	Z-L-Val	d
Trp	63.68	64.14	Z-L-Val	d
Tyr	51.70	51.52	Z-L-Val	d
Val	56.73	55.36	Z-L-Val	d

a. Isocratic 63% solvent B; b. isocratic 40% solvent B; c. isocratic 100% solvent B; d. isocratic 10% solvent B in 15 min and linear gradient (A:B = 90:10 (15 min) → 50:50 (60 min)).

The 38 synthesised dipeptides containing Z-L*-Val as the chiral auxiliary were analysed by ERMS, and their E_{50} values were obtained (Supplementary Fig. S1). It seemed that a rule might exist for the diastereoselection in the series of amino acids. The results showed that L*D-isomers were stable, with larger E_{50} values than L*L-isomers, except for the cases of Asn and Gln. Steric hindrance was considered as a basis for the differences in E_{50} values. Thus, we

initially thought that all sets of dipeptides might show the same tendency. In the case of Asn and Gln, it was found that the L*L-isomers were more stable than the L*D-isomers. This seemed to be contradictory. As shown by the results of the theoretical calculations of 1(*R* and *S*)-methylpropylamine containing Z-(*S*)-Pro, the discrimination is considered to be a result of steric factors. In this series of dipeptides, the only difference in the structures of the pairs of diastereomers is the orientation of the side chain of the amino acids. Thus, it is reasonable that the majority of dipeptides showed the same tendency. The different results obtained for Asn and Gln can be explained by a report that the amide in the side chain forms intramolecular hydrogen bonds[1], which may restrict conformational freedom and makes the orientation of the carboxyl carbonyl group different from the others, affecting the stability of the ions. Thus, steric hindrance

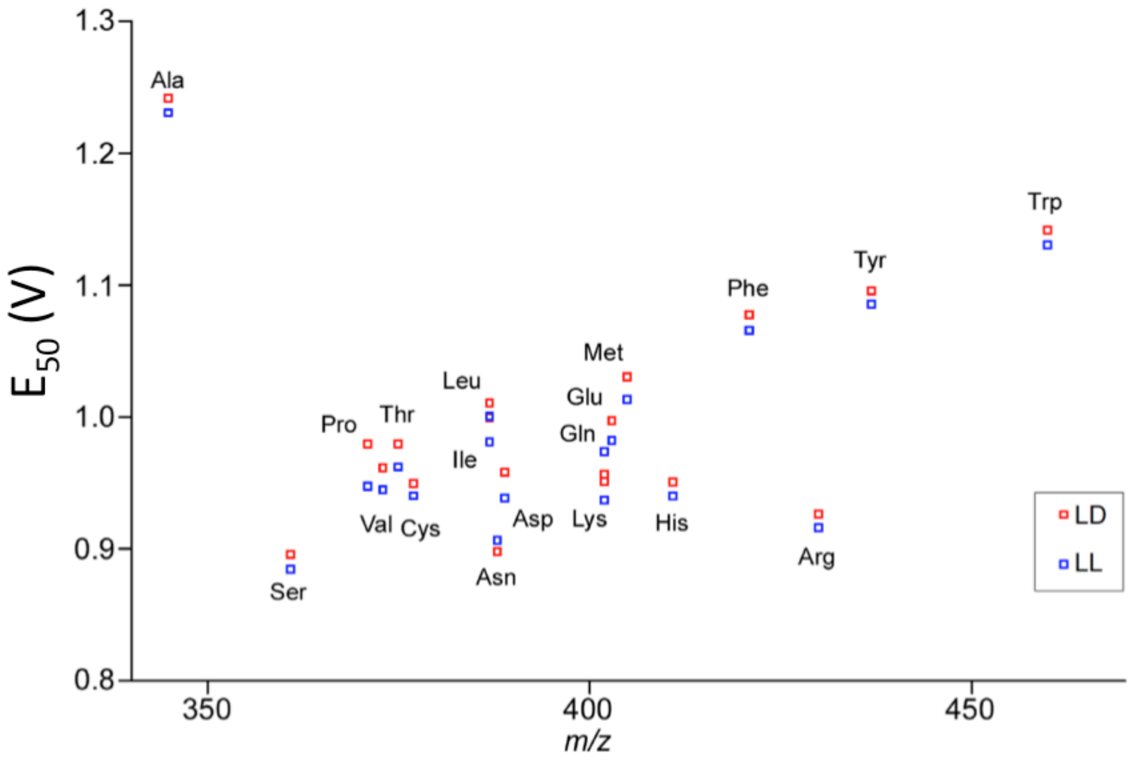

Supplementary Fig. S1. ERMS-based discrimination of all amino acids containing Z-L-Val as the auxiliary.

was greater in the L*D-isomers, and dipeptides derived from Asn and Gln had a reverse relationship in E_{50} values compared to the other 17 pairs of amino acids. All of our experimental data strongly indicated that the analysis of the decay process of the sodiated molecules could generally resolve diastereomers.

Typical synthesis of a mixture consisting of a set of amino acids containing Z-L-Val as the chiral auxiliary. Triethylamine (0.5 mL) was added to a solution containing a series of amino acids (ca 0.015 μmol), namely L-Ile (1.4 mg), L-Phe (1.7 mg) and L-Pro (2.0 mg), and Z-L-Val (12.3 mg, 0.035 μmol) dissolved in 1,4-dioxane–water (1:1, 1 mL). The mixture was stirred at room temperature for 23 h. The mixture was poured into ethyl acetate and washed with 1*N* HCl. The organic layer was dried with $MgSO_4$ and evaporated to dryness. A methanolic solution of the resulting residue was directly used for the mass spectrometric analysis.

Note for statistical analysis. Analysis of the decay curve obtained for a series of data, while the end-cap radio frequency (r.f.) amplitude was raised by 0.02 V increments, was carried out as follows, where (*S*)-1-methylpropylamine (**6**) containing Z-(*S*)-Pro as a chiral auxiliary is described as an example. In an ordinary data analysis, multiple product ions are observed, and all of the ions, including the precursor ion, are normalised and shown as percentages. However, in this case no product ion was observed, and thus normalisation could not be carried out. For this reason, a global analysis of a series of data sets for the individual experiments was carried out. One of the concerns was that precise control of the number of trapped precursor ions to be subjected to CID is generally not possible. In the current experiments, the numbers of ions ranged from 1.61×10^6 to

1.82×10^6. The collision gas pressure was set to 6.0×10^{-6} mbar during the CID process. When a large excess of He gas is present, the reaction kinetics of the fragmentation reaction producing a series of product ions and the dissociation of adducted metals can be discussed. Here, the quantities of the precursor ions reached zero at an r.f. amplitude of 1.42 through 1.44 V in all experiments, which indicated that an excess amount of collision gas, He, was present, regardless of the amount of precursor ions in the individual experiments. It should be noted that the trapped precursor ions subjected to the CID conditions remained stable in an electric field. Based on the above considerations, statistical analysis of the ERMS data obtained for a series of ions was carried out as follows.

Data were obtained from five continuous experiments where individual data set consisted of 51 data corresponding to an applied r.f. amplitude (0.50–1.50 V). Global analysis of these data were first carried out using Boltzmann non-linear regression curve fitting, where individual regression curves share an E_{50} (b) value the slope factor (c) based on an assumption that these data should share a common values. After this first analysis, four out of five data sets were selected according to the R^2 values. The obtained R^2 values were 0.9888, 0.9902 9901 0.9916, 0.9634, and 0.9758. Four data sets except for the data giving poor R^2 value were again subjected to global analysis to obtain curves with shared slope factor, resulting in good R^2 values of 0.9885, 0.9902, and 0.9917, and 0.9760. The obtained E_{50} values of these data obtained from second sigmoidal curve fitting, namely 0.9641, 0.9575. 0.9562, and 0.9640, were plotted together with standard error to the mean for comparison with data obtained for its diastereomer, which were further subjected to a Mann-Whitney U test.

Reaction analysis of Z-L-Val-Pro. While obtaining the E_{50} values of the diastereomers of Z-L-Val-Pro under low-energy CID conditions, a series of product ions were obtained (Fig.

1a). All of the observed product ions can be assigned, as shown in Supplementary Fig. S2, where the loss of the Pro residue leading to the formation of an ion with *m/z* 274 was the major pathway.

Consideration on day-to-day variation. Since exact CID conditions cannot be reproduced, obtained break down curves in ERMS shift reflecting the reaction conditions, and thus day-to-day variation is fundamental problem in such analysis. For example, when one tries to obtain absolute E_{50} values for compounds, the values shift over time that makes comparison impossible. However, the relative tendency in differences of E_{50} values between diastereomers

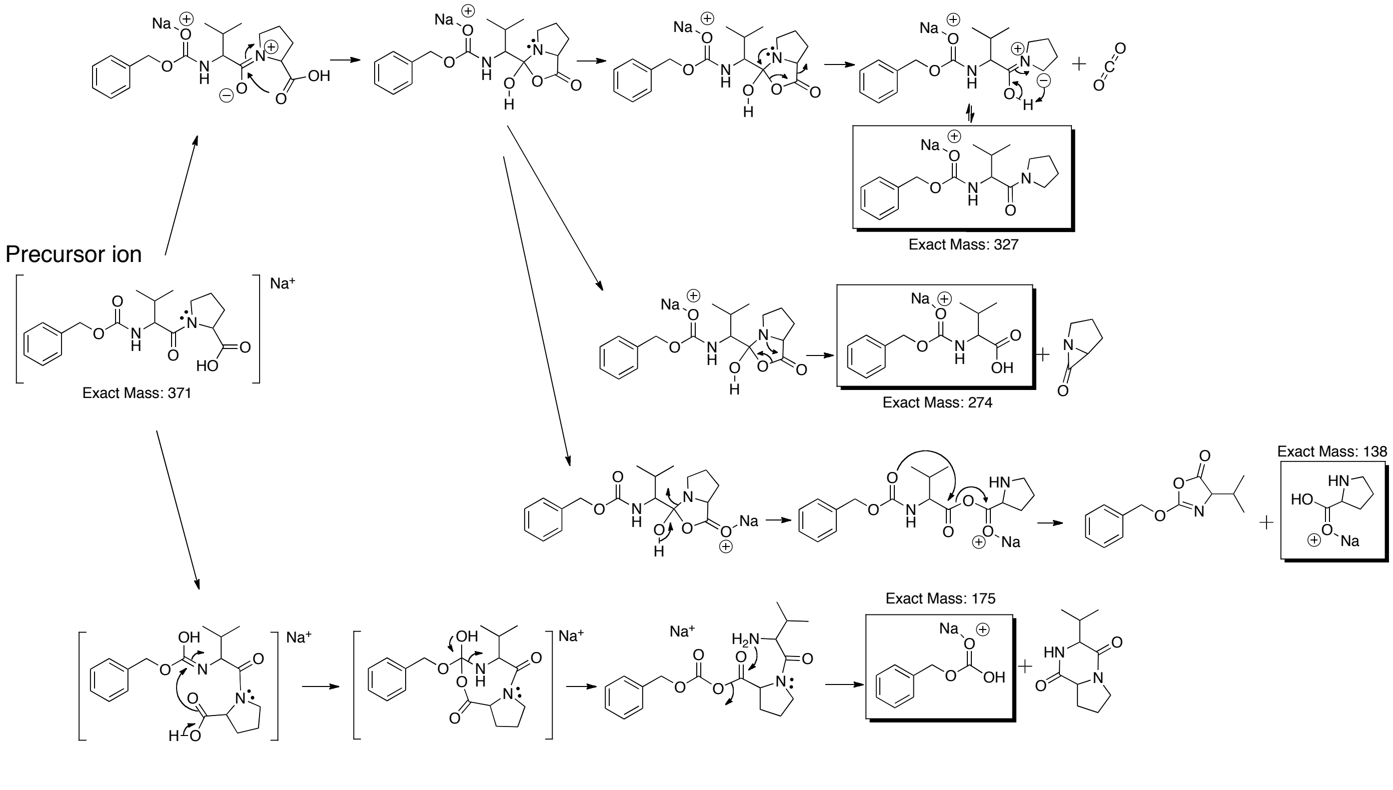

Supplementary Fig. S2. Postulated fragmentation pathways for the enantio and diastereopairs of Boc-L-Val-L-Pro dissociation. The observed ion species with exact mass numbers are highlighted in the box.

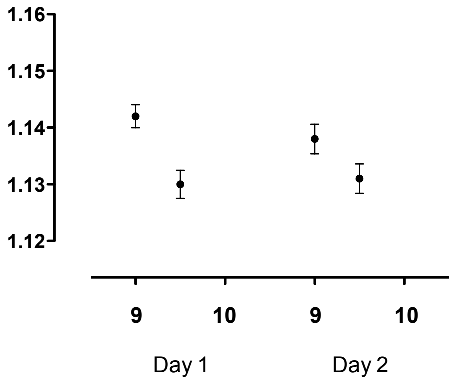

Supplementary Fig. S3. E_{50} values of sodiated compounds **9** and **10** obtained several days apart. Mean and standard error of the mean obtained for the curve fitting was shown.

remains indicating that the experiments are reproducible in terms of obtaining relative difference (Supplementary Fig. S3).

The collision gas pressure and CID time, in addition to the resonant excitation parameters, control the fragmentation reactions and dissociation of adducted metal ion. Therefore, examining the reaction conditions by changing these MS parameters may improve the E_{50} difference. Furthermore, the use of external energy reference might be possible enabling normalisation of obtained E_{50} values.

References

1. A. L. Heaton & P. B. Armentrout, Thermodynamics and mechanism of the deamidation of sodium-bound asparagine. *J. Am. Chem. Soc.* **130,** 10227-10232 (2008).
